# Tonsillar granuloma associated with hypogammaglobulinemia

**DOI:** 10.1186/s13223-020-00441-1

**Published:** 2020-05-29

**Authors:** Aleksi Laajala, Outi Kuismin, Mikko Tastula, Leena Tiitto, Saila Kauppila, Anna Salo, Pirjo Åström, Antti Nissinen, Virpi Glumoff, Mikko R. J. Seppänen, Timo Hautala

**Affiliations:** 1 Department of Otorhinolaryngology and Head and Neck Surgery, Oulu University Hospital, Oulu, Finland; 2 PEDEGO Research Unit, Medical Research Center, University of Oulu, Oulu, Finland; 3 Department of Clinical Genetics, PEDEGO Research Unit, Medical Research Center, Oulu University Hospital and University of Oulu, Oulu, Finland; 4 Respiratory Medicine, Research Unit of Internal Medicine, University of Oulu and Medical Research Center Oulu, Oulu University Hospital, Oulu, Finland; 5 Department of Pathology, Cancer Research and Translational Medicine Research Unit, University of Oulu and Oulu University Hospital, Oulu, Finland; 6 Department of Radiology, Oulu University Hospital, Oulu, Finland; 7 Research Unit of Biomedicine, University of Oulu, Oulu, Finland; 8 Rare Disease Center and Pediatric Research Center, Children and Adolescents, Adult Immunodeficiency Unit, Inflammation Center, University of Helsinki and HUS Helsinki University Hospital, Helsinki, Finland; 9 Department of Internal Medicine, Oulu University Hospital, Oulu, Finland

**Keywords:** Granuloma, Hypogammaglobulinemia, Rituximab, Sarcoidosis

## Abstract

**Background:**

Rare tonsillar granulomas may be caused for example by infections, malignancies or sarcoidosis. Granulomas also occur in inborn errors of immunity (IEI) such as common variable immunodeficiency (CVID) with B cell maturation defects and hypogammaglobulinemia. CVID shares various features with sarcoidosis and drug-induced secondary hypogammaglobulinemia; careful consideration of differential diagnosis between these conditions is warranted.

**Case presentation:**

A 29-year-old female with epilepsy developed dysphagia, dyspnea and impaired exercise tolerance. Obstruction caused by swollen lingual tonsil and edema in the epiglottis and arytenoid mucosa were found. Lingual tonsil and epiglottis biopsies displayed non-necrotizing granulomas. There was no evidence of viral, bacterial, mycobacterial or fungal infections. Chest X-ray, computerized tomography of chest and ultrasound of neck and abdomen remained unremarkable. Positron emission tomography/computed tomography (PET/CT) showed laryngeal enhancement. Empiric antimicrobials combined with prednisolone were insufficient to control her disease. In immunological evaluation, the patient had normal counts of B and T cells. Proportions of CD27^+^ memory B cells (30.3%) and IgD^−^IgM^−^CD27^+^ switched memory B cells (7.2%; normal range 6.5–29.2%) were normal. Percentage of activated CD21^low^ B cells was high (6.6%; normal range 0.6–3.5%). IgG (3.5 g/L; normal range 6.77–15.0 g/l) and all IgG subclass concentrations were low. Anti-polysaccharide responses were impaired, with 3/10 serotypes reaching a level of 0.35 µg/ml after immunization with Pneumovax^®^. The findings were consistent with hypogammaglobulinemia resembling CVID, possibly secondary to antiepileptic medication. Her dyspnea and dysphagia responded favorably to subcutaneous IgG and rituximab.

**Conclusions:**

Tonsillar granulomas can be the presenting and only clinical feature of B cell deficiency, highlighting the diversity of symptoms and findings in primary or secondary immunodeficiencies.

## Background

Tonsillar granulomatous inflammation is rare, most commonly caused by tuberculosis or sarcoidosis. Rarely, Hodgkin’s lymphoma, toxoplasmosis, fungal infection and squamous cell carcinoma are associated with pharyngeal granulomas [1, 2]. Granulomatous inflammation is frequently seen in common variable immunodeficiency (CVID), a heterogeneous immune defect characterized by aberrant B cell maturation, hypogammaglobulinemia, failure of specific antibody production, susceptibility to infections, and variable comorbidities [3–5]. Approximately 10–20% of CVID patients suffer from granulomatous inflammation, most commonly affecting lymph nodes and lungs [6, 7]. Differential diagnosis of secondary causes, such as drug induced mechanisms, must be considered whenever CVID or B cell maturation defect is suspected. In addition, distinguishing granulomatous CVID from sarcoidosis remains challenging, as recently reviewed by Ameratunga et al. [8]. Our patient presented with unusual granulomatous inflammation of the lingual tonsil and epiglottis causing dysphagia and airway obstruction; her condition shares features with primary and drug-induced B cell deficiency as well as sarcoidosis.

## Case presentation

A 29-years-old female experienced an episode of mild upper respiratory tract infection followed by a slowly developing dysphagia and dyspnea. This led to impaired exercise tolerance lasting several months, with recent subacute exacerbation. There was no significant history of travel or exposure to infectious agents. She had not suffered from fever or other acute or chronic infectious symptoms. Her palatine tonsils had been removed in childhood. She was diagnosed with epilepsy at age 17 and had been free of epileptic symptoms for over 5 years with levetiracetam (500 mg two times per day) and lamotrigine (150 mg two times per day). Her family history was unremarkable.

**Fig. 1:**
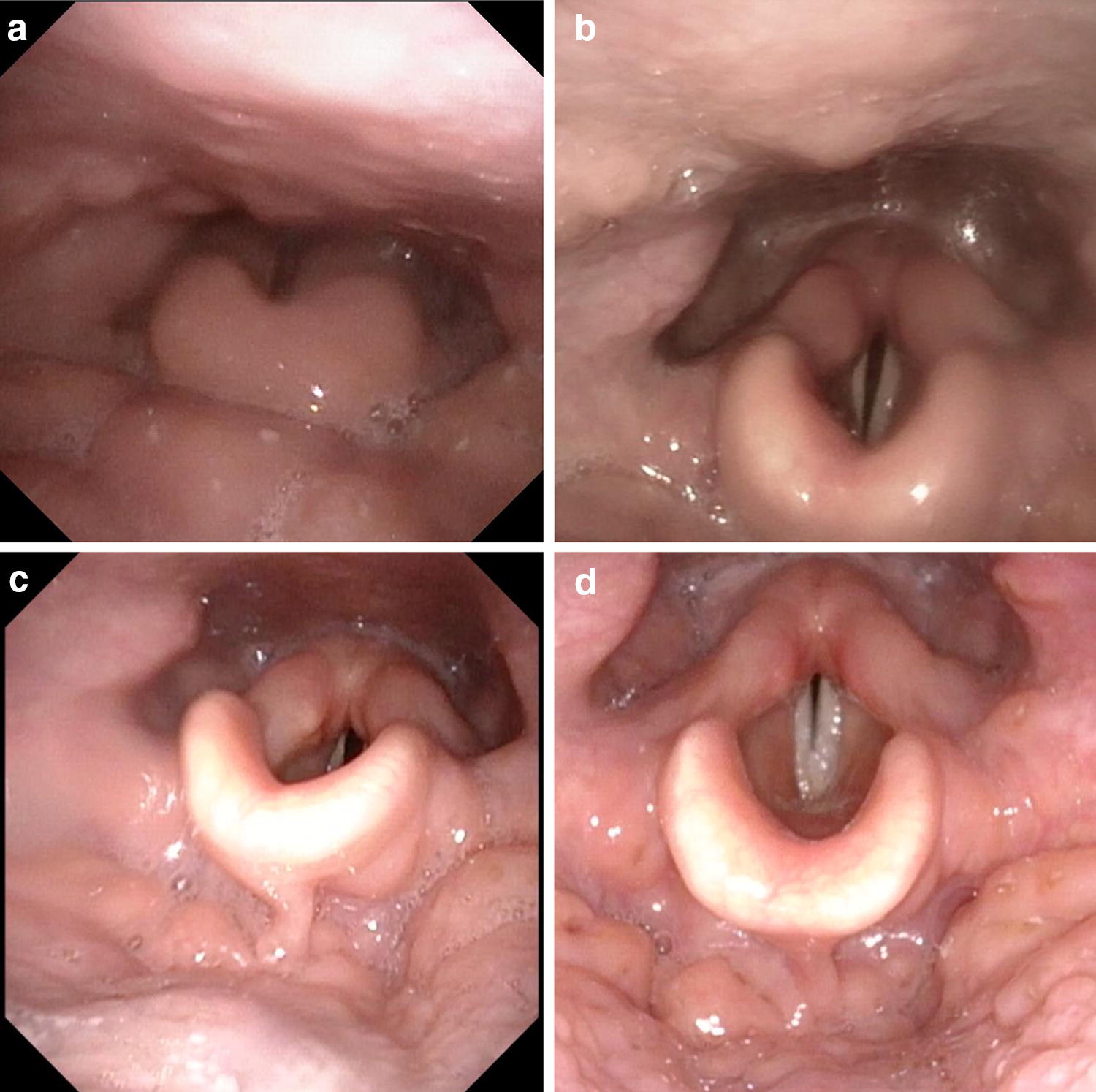
Fiberoptic findings in primary situation (**a**) and a modest improvement in edema and symptoms during prednisolone 60 mg/days treatment (**b**). Significant improvement in edema and symptoms 2 weeks after rituximab (**c**). Seven months after rituximab treatment, the patient was free of symptoms (**d**)

She spoke with hoarse voice, without signs or findings that would have suggested systemic involvement. There were no signs of generalized mucosal disease. Fiberoptic examination showed swelling of the lingual tonsil, epiglottic and arytenoid mucosa, causing airway obstruction (Fig. 1a). C-reactive protein concentration and blood sedimentation rate were low, and anti-nuclear, anti-neutrophil, anti-glomerular basement membrane, anti-myeloperoxidase, anti-proteinase 3, tissue transglutaminase and cyclic citrullinated peptide antibodies were negative. Thyroid function was normal and thyroid peroxidase antibodies were 34 IU/ml (normal value < 60 IU/ml). Plasma parathyroid hormone (39 ng/l; normal 18–80 ng/l) and serum vitamin D-25 (77 nmol/l; normal > 50 nmol/l) were normal. No evidence for acute or chronic viral, bacterial, mycobacterial or fungal infections, including hepatitis B and C, human immunodeficiency virus and tularemia, was found.

**Fig. 2:**
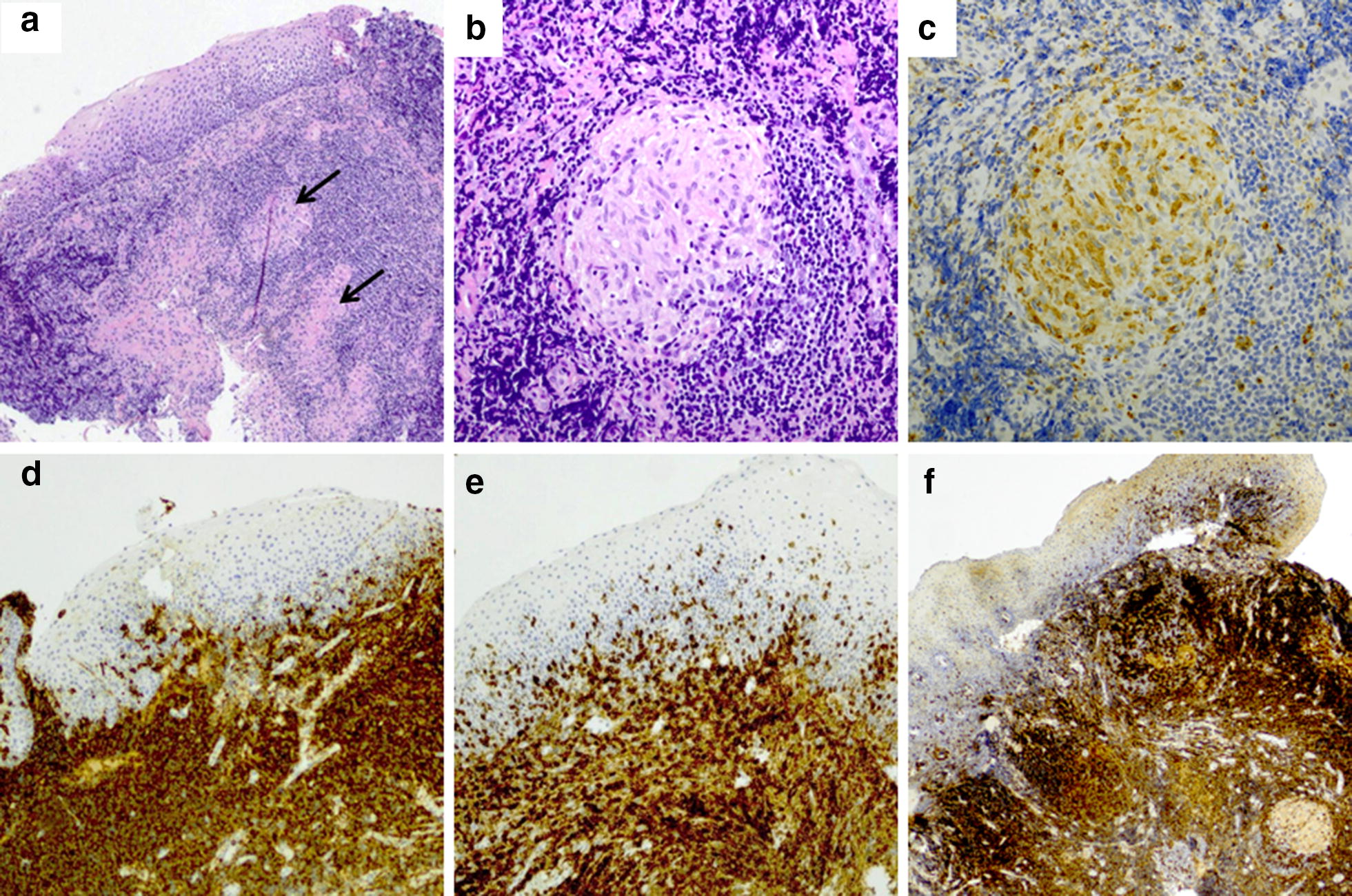
Histological findings (**a**, **b**) showing granulomatous reaction in lingual tonsil with CD68 (Kp-1) positive epitheloid histiocytes by immunohistochemistry (**c**). Strong CD20-immunopositivity in B-lymphocytes (**d**). Most CD3-positive (**e**) T-lymphocytes showed also CD4-positivity (**f**)

Due to her swollen lingual tonsil and laryngeal mucosa causing airway obstruction, she was hospitalized and received empiric cefuroxime (1.5 g thrice daily) and methylprednisolone (75 mg once daily, height 173 cm, weight 63 kg, prednisolone dose 1.2 mg/kg) intravenously. After a transient positive response, she was discharged from the hospital after 6 days. Soon after, she was readmitted to due to a rapid reoccurrence of the symptoms. A modest positive response was seen after per oral clindamycin and oral prednisolone (60 mg/days) with dose reduction for 14 days. Lingual tonsil and epiglottis were biopsied. Histology showed granulomatous reaction in lingual tonsil with CD68^+^ (Kp-1) epithelioid histiocytes (Fig. 2). The CD20^+^ B lymphocyte count was increased while Pax-5^+^ positive B lymphocyte count was normal. CD138^+^ plasma cells were scarce, and they showed polytypic kappa- and lambda light chain expression. The number of CD3^+^ T cells was normal, and most T cells were CD4^+^. Eosinophils were practically absent and well-formed follicles were not seen. Epstein–Barr virus in situ hybridization (EBER), p16, cytomegalovirus and mycobacteria were negative. Morphology, immunohistochemical staining, serum electrophoresis and bone marrow aspirate did not raise suspicion of lymphoid neoplasia, thus, no clonality studies were done.

**Fig. 3:**
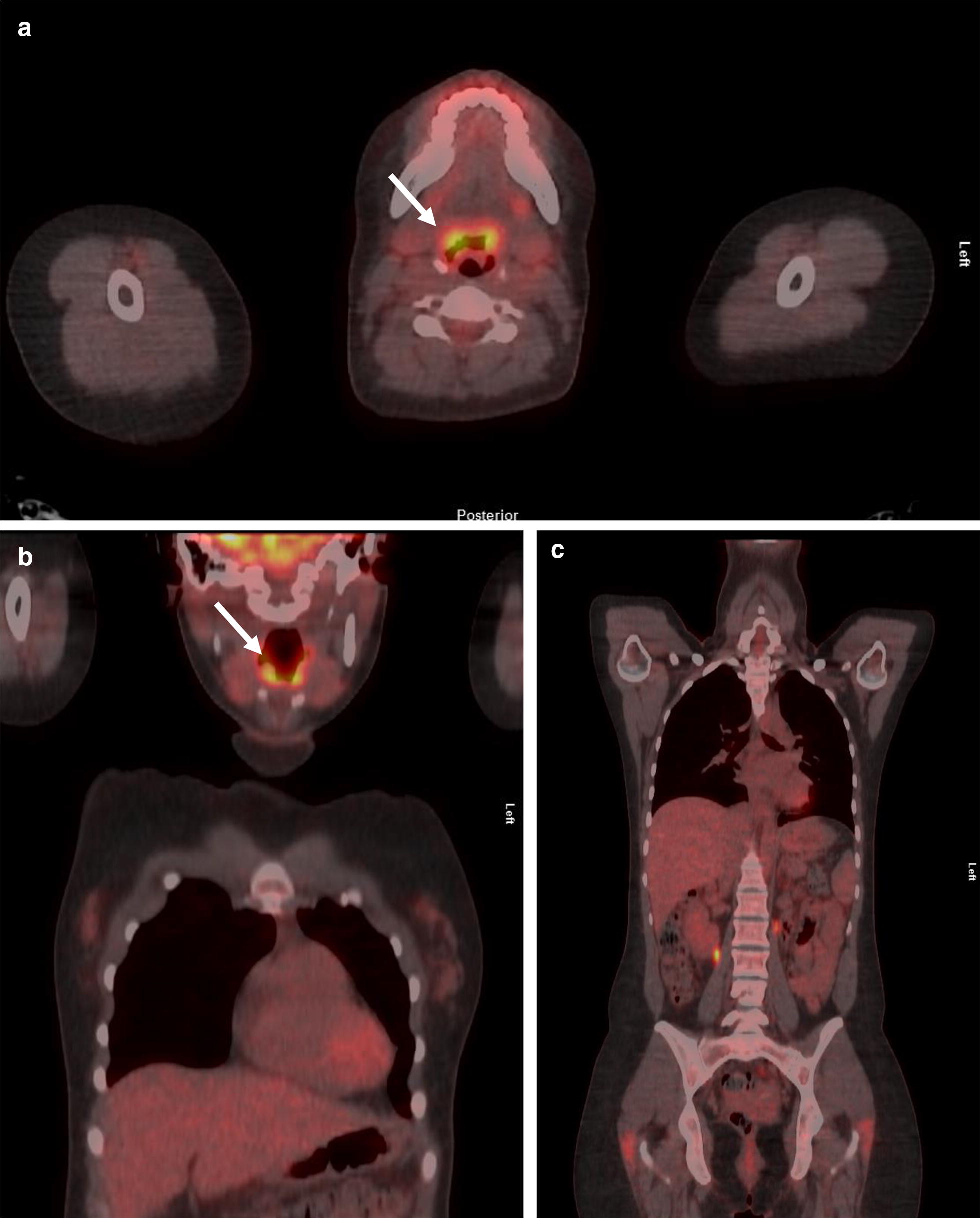
Positron emission tomography—computed tomography showed increased activity (arrows) in the lingual tonsil (**a**, **b**) without evidence of widespread sarcoidosis (**c**)

Due to granulomas, sarcoidosis was considered. However, chest X-ray, computerized tomography of chest and ultrasound of neck and abdomen were unremarkable. Positron emission tomography/computed tomography (PET/CT) showed increased fluorodeoxyglucose (FDG) uptake in the lingual tonsil, without evidence of widespread sarcoidosis (Fig. 3). No other pathological FDG foci were found. Focal physiological FDG accumulation was seen in ureters on both sides of the spinal column. This is a common finding due to ureteral peristalsis and pooling of radiotracer in the recumbent patient. The patient had no neurological symptoms and brain magnetic resonance imaging was normal. Serum angiotensin converting enzyme (ACE) was within normal range (11–20 U/l; reference range 9–65 U/l) while daily urine calcium output was normal or slightly elevated (5.0–6.31 mmol; reference range 1.2–5.5 mmol).

Subsequently, an unusual presentation of primary or secondary immunodeficiency was considered. There was no consanguinity; the patient or her family had no history of repeated infections or autoimmunity. White cell, lymphocyte, and B, T and NK lymphocyte subset counts were within normal limits. CD19^+^ B cell count was 153 × 10^9^/l (normal range 80–616 × 10^9^/l). Proportions of memory CD27^+^ B-cells (30.3%) and IgD^−^IgM^−^CD27^+^ switched memory B cells 7.2%; (normal range 6.5–29.2%) were normal and the percentage of activated CD21^low^ B cells was high (6.6%; normal range 0.6–3.5%). Percentages of various CD3^+^CD4^+^ and CD3^+^CD8^+^ T cell subsets appeared normal. Serum total IgG (3.5 g/l; normal range 6.77–15.0 g/l) and all IgG subclass concentrations were low: IgG1 2.45 g/l (normal range 4.9–11.4 g/l), IgG2 0.91 g/l (normal range 1.5–6.4 g/l), IgG3 0.18 g/l (normal range 0.2–1.1 g/l) and IgG4 0.02 g/L (normal range 0.08–1.4 g/l). Anti-polysaccharide response to Pneumovax^®^ were impaired, with only 3/10 serotypes reaching a level of 0.35 µg/ml after immunization. However, serum IgA (0.90 g/l; normal range 0.52–4.02 g/l) and IgM concentrations (0.57 g/l; normal range 0.47–2.84 g/l) were normal. Dihydrorhodamine test excluded chronic granulomatous disease. These findings were thus consistent with either granulomatous CVID or most likely secondary drug-induced hypogammaglobulinemia with a novel clinical presentation [3–5]. Genetic analysis (Blueprint Genetics, Primary Immunodeficiency Plus panel. https://blueprintgenetics.com/tests/panels/immunology/primary-immunodeficiency-panel/) did not find known mutations causative of CVID-like or other primary immunodeficiencies.

Due to symptomatic upper airway obstruction, dysphagia, lack of evident infection and unsatisfactory response to prednisolone treatment and possible granulomatous CVID, the patient received immunoglobulin replacement and rituximab therapy (Mabthera^®^ 100 mg, 200 mg and 500 mg on three consecutive days combined with 100 mg hydrocortisone and 1 g paracetamol), with a favorable response within 2 weeks accompanied by significant improvement in dysphagia and respiratory symptoms. Seven months later, her exercise capacity was good. She has remained asymptomatic for over 16 months after rituximab treatment. Her fiberoptic pharyngeal findings improved (Fig. 1). She continues to receive subcutaneous immunoglobulin replacement with serum IgG levels in the range of 10 to 12 g/l.

## Discussion and conclusions

Our patient shares clinical and laboratory features with granulomatous CVID, with mild elevation in daily urine calcium. She lacks the most common and generally accepted sarcoidosis findings while plasma IgG levels and anti-polysaccharide antibody levels after Pneumovax^®^ were low [9–11]. Low IgG concentration and inability to respond to a vaccine combined with B cell depletion therapy in serious airway involvement was thought to warrant IgG replacement. Although our patient’s condition resembles “possible CVID” [3–5], normal blood IgA and CD4 levels are not typical of granulomatous CVID [6, 7]. She had been on antiepileptic treatment without obvious side-effects for years. Clozapine, carbamazepine, phenytoin, lamotrigine and levetiracetam use may rarely be associated with the development of subsequent hypogammaglobulinemia [12–14]. Her condition may have developed secondary to antiepileptic treatment. These observations highlight the importance of potential drug-induced mechanisms in B-cell deficiencies.

Whether our patient suffers from a novel and unusual presentation of primary or secondary hypogammaglobulinemia remains to be determined. Though granulomatous lesions in CVID are common, to our knowledge tonsillar and epiglottic lesions have never been described. Good rituximab responses are commonly seen in CVID; limited rituximab experience in select sarcoidosis cases with poor response to primary medications has been reported. Based on our experience, we suggest that the diagnosis of primary or secondary immunological causes should be considered in tonsillar and/or epiglottic granulomas (Fig. 2).

## Data Availability

Not applicable
